# Prevalence, associated factors, and outcomes of behavioural and psychological symptoms of dementia in acute hospitals: a systematic review and meta‐analysis

**DOI:** 10.1002/alz70857_096052

**Published:** 2025-12-24

**Authors:** Kanthee Anantapong, Aimorn Jiraphan, Warut Aunjitsakul, Katti Sathaporn, Nisan Werachattawan, Teerapat Teetharatkul, Pakawat Wiwattanaworaset, Nathan Davies, Elizabeth Lesley Sampson

**Affiliations:** ^1^ Prince of Songkla University, Hat Yai, Songkhla, Thailand; ^2^ Queen Mary University of London, London, London, United Kingdom; ^3^ Royal London Hospital, East London NHS Foundation Trust, London, United Kingdom; ^4^ Barts Health NHS Trust Queen Mary University of London, London, London, United Kingdom

## Abstract

**Background:**

Acute hospital care can be complicated by behavioural and psychological symptoms of dementia (BPSD), yet the evidence in this setting is diverse. Our study aimed to assess BPSD prevalence in acute hospitals and investigate associated factors, management strategies, and outcomes (PROSPERO: CRD42023406294).

**Method:**

We performed a systematic review and meta‐analysis, searching Cochrane Library, MEDLINE, and PsycINFO for studies reporting BPSD prevalence in older dementia patients during acute hospital stays (until 5 March 2024). We employed independent double‐review processes for study selection and data extraction. Twelve BPSD symptoms were analysed using the Neuropsychiatric Inventory Questionnaire (NPI) and Behavioural Pathology in Alzheimer's Disease (BEHAVE‐AD) scales. We summarised risk factors, treatments, and outcomes, and conducted meta‐analyses to synthesise findings.

**Result:**

From 15,101 initial records, we included 30 articles representing 23 studies, encompassing 109,805 older patients (range: 42‐53,156) in acute hospitals. Study quality was predominantly moderate (*n* = 12) to poor (*n* = 17). Meta‐analysis revealed a pooled BPSD prevalence of 60% (range: 8‐95%, 95% CI=43‐78%) among older inpatients with dementia (*N* = 11 studies). Subgroup analysis showed varying BPSD prevalence based on assessment tools (BEHAVE‐AD=85%, NPI=74%, Others=40%). A sensitivity analysis excluding one study with delirium screening criteria yielded stable results. Prevalent BPSD included aggression/agitation (39%), sleep disturbances (38%), eating problems (36%), and irritability (32%). BPSD were associated with delirium, pain, increased use of uncomfortable interventions, psychotropic medication use, and heightened caregiver stress. Suboptimal patient‐staff interactions and inadequate discharge planning were linked to frequent emergency visits and readmissions.

**Conclusion:**

Our findings underscore the high prevalence and significant impact of BPSD in acute care settings. We recommend that healthcare systems develop targeted BPSD management strategies, enhance staff training, improve communication with caregivers, and implement comprehensive discharge planning. Further research should focus on developing and evaluating interventions specifically designed for BPSD in acute hospital environments, aiming to enhance patient outcomes and alleviate caregiver burden.

